# Impact of Prior Abdominal Surgery on Total Laparoscopic Hysterectomy vs Total Abdominal Hysterectomy: A Retrospective Cohort Study

**DOI:** 10.7759/cureus.79151

**Published:** 2025-02-17

**Authors:** Yaser M Almonla, Khalid Akkour, Miyad Khazna, Salim M Abduljawad

**Affiliations:** 1 College of Medicine, Alfaisal University, Riyadh, SAU; 2 General Practice, Kingdom Hospital, Riyadh, SAU; 3 Gynecologic Oncology, King Khalid University Hospital, Riyadh, SAU

**Keywords:** abdominal surgeries, laparoscopic, laparoscopic-assisted vaginal hysterectomy, s: hysterectomy, total abdominal

## Abstract

Objective

This study aims to compare the outcomes of total laparoscopic hysterectomy (TLH) and total abdominal hysterectomy (TAH) in patients with and without prior abdominal surgery, focusing on key factors such as blood loss, operative time, complication rates, and hospital stay. The primary aim is to assess the impact of previous abdominal surgery on surgical outcomes.

Methods

A retrospective cohort study was conducted at a private hospital in Saudi Arabia between January 2019 and July 2024. The study included patients who underwent TLH or TAH, with or without prior abdominal surgery, such as cesarean sections (CSs), appendectomies, cholecystectomies, and hernia repairs. A total of 163 procedures were performed by a single surgeon. Patients aged 18-70 years were included, while cases in which a hysterectomy was immediately followed by an elective CS were excluded to prevent bias in operative time and blood loss measurements. Surgical outcomes were then compared between groups.

Results

A total of 151 patients were included, with 56 undergoing TLH and 95 undergoing TAH. Prior abdominal surgery was present in 24 (42.9%) TLH patients and 33 (34.7%) TAH patients. Median blood loss was significantly lower in the TLH group (200 mL, IQR: 100-300) compared to the TAH group (300 mL, IQR: 200-500) (p = 0.00). The median hospital stay was also shorter for TLH patients (two days, IQR: 1-3) than for TAH patients (two to three days, IQR: 2-3) (p = 0.00). There was no significant difference in operative time between TLH (98.5 minutes, IQR: 83.25-116.5) and TAH (95 minutes, IQR: 76-120) (p = 0.633). Complications occurred in four (7.1%) TLH patients and seven (7.4%) TAH patients, with bladder injuries reported in two patients from each group and bowel injuries in three TAH patients. The majority of patients in both groups experienced no complications (92.9% in TLH and 92.6% in TAH).

Conclusions

TLH provides significant advantages over TAH, including reduced blood loss and shorter hospital stays, even in patients with prior abdominal surgeries. It remains a safe and effective option when performed by experienced surgeons. However, the choice between TLH and TAH should be individualized, taking into account factors such as the extent of adhesions, surgical expertise, and patient health. Further research is recommended to optimize outcomes in complex cases.

## Introduction

Hysterectomy is one of the most common procedures in gynecological practice, performed to treat various benign and malignant conditions such as fibroids, abnormal uterine bleeding, endometriosis, and gynecological cancers. Over the years, surgical techniques have advanced to minimize patient harm and reduce recovery time. While total abdominal hysterectomy (TAH) has traditionally been the preferred approach due to its advantages of providing better exposure to the surgical field and easier access to pelvic structures, total laparoscopic hysterectomy (TLH) has gained popularity in recent years. Its minimally invasive nature offers benefits such as reduced postoperative pain, earlier ambulation, shorter hospital stays, and lower infection rates [1,2].

A key challenge in TLH is the presence of adhesions - scar tissue that forms between tissues and organs after surgery - which can complicate subsequent procedures by obscuring the surgical field and increasing the risk of injury to adjacent structures. This is particularly relevant in TLH, where poor visibility and limited access can turn a routine surgery into a complex one [3]. Consequently, prior abdominal surgeries, including cesarean sections (CSs), appendectomies, and cholecystectomies, must be carefully considered when assessing the risks of TLH [4].

CSs are among the most common surgeries worldwide and are a frequent comorbidity in women undergoing hysterectomy. The incidence of adhesions after CS ranges from 22% to 46%, increasing with multiple prior CSs [5]. Adhesions involving the bladder and uterus can complicate laparoscopic entry, raising the risk of complications such as bladder injury. While some studies suggest that TLH remains a safe option for these patients, the technical difficulty of the procedure increases, often resulting in longer operative times and a higher likelihood of complications [6]. In some cases, severe adhesions necessitate conversion to an open procedure. Lim et al. [7] reported that while TLH is feasible in patients with prior CS, the presence of adhesions significantly increases surgical complexity, requiring meticulous technique and caution.

Adhesions and anatomical changes are not exclusive to patients with prior CS. Other abdominal surgeries, including appendectomy, cholecystectomy, and hernia repair, can also lead to postoperative adhesions and structural alterations [8]. Therefore, patients with such surgical histories require thorough preoperative planning, and in some cases, conversion to TAH may be necessary due to intraoperative complications [9,10].

Although TAH provides superior surgical access compared to TLH, it is associated with longer recovery times, increased postoperative pain, higher rates of wound complications, and prolonged hospital stays [11]. Thus, the decision between TLH and TAH in patients with prior abdominal surgeries must balance the benefits of each approach against surgical complexity, patient health status, and overall prognosis. A retrospective study by Koroglu et al. [12] found that prior abdominal surgery increased the difficulty of TLH, leading to longer operative times and a higher rate of conversion to open surgery.

If TLH is attempted but adhesions or anatomical changes hinder the procedure, conversion to TAH may be required. However, research suggests that conversion should not be viewed as a failure but rather as a means of ensuring patient safety. Seo et al. [5], in their analysis of 161 TLH cases, emphasized that in the presence of severe adhesions, switching to an open procedure is necessary to prevent serious complications. Examples of such complications include bladder and bowel injuries, highlighting the importance of conversion when required to safeguard patient outcomes.

These factors raise the critical question of whether TLH or TAH is preferable for patients with a history of abdominal surgery. Surgeons must carefully weigh the advantages and disadvantages of each approach, taking into account the extent of adhesions, the patient’s surgical history, and the feasibility of minimally invasive surgery. A study by Müller et al. [2] suggests that TLH can be successfully performed in patients with prior abdominal surgeries, provided the surgeon has sufficient expertise and patient selection is appropriate.

This study aims to assess the impact of prior abdominal surgeries on TLH compared to TAH. By evaluating surgical outcomes in this patient population, the study seeks to provide valuable insights for clinicians in selecting the most appropriate approach for women with a history of abdominal surgery. The findings may help healthcare providers minimize risks associated with complex surgical procedures and improve patient outcomes.

## Materials and methods

Study design and setting

This retrospective cohort study was conducted at a private hospital in Saudi Arabia and included all cases of TLH and TAH performed between January 2019 and July 2024. Women aged 18-70 years who underwent either TLH or TAH, with or without prior abdominal surgery - including CSs, appendectomies, cholecystectomies, hernia repairs, and abdominoplasty - were included. Cases in which a hysterectomy was immediately followed by an elective CS were excluded to prevent bias in operative time and blood loss measurements.

Clinical characteristics and comorbidities

To ensure group comparability and account for confounding factors, we collected data on patient clinical characteristics, including age, BMI, and comorbidities. The Charlson Comorbidity Index (CCI) was used to quantify the overall burden of comorbidities for each patient. By assigning weighted scores to specific conditions such as diabetes, cardiovascular disease, and chronic kidney disease, the CCI helps predict the risk of mortality and morbidity. This approach allowed us to control for the impact of comorbidities on surgical outcomes, including operative time, blood loss, complications, and hospital stay.

Data collection

Data were retrospectively collected from hospital records, focusing on surgical outcomes such as operative time, complication rates, and conversion to open surgery. Additionally, information on the type of previous abdominal surgery and clinical characteristics, including age, BMI, and CCI scores, was recorded.

Ethical considerations

This study strictly adhered to ethical guidelines and best practices. Before initiation, the study protocol was submitted to the hospital’s institutional review board to ensure ethical compliance. Additionally, the study followed Good Clinical Practice guidelines, prioritizing the protection of participants’ rights, safety, and welfare throughout the research process. Patient identification details and all related information were safeguarded to maintain confidentiality. The raw data used in the study did not contain any personally identifiable information, and patient participation had no impact on their treatment.

Statistical methods

The statistical analysis in this study focused on comparing surgical outcomes between TLH and TAH. The following statistical techniques were applied:

Descriptive Statistics

Frequencies and percentages: These were used to summarize categorical variables, including the type of prior abdominal surgeries, the presence or absence of complications, and the reasons for readmission.

Medians and IQRs: These were applied to summarize continuous variables such as blood loss, operative time, and length of hospital stay. Medians were chosen over means to account for non-normal data distributions and to minimize the influence of outliers.

Comparative Analysis

Mann-Whitney U test: This nonparametric test was used to compare continuous variables, such as blood loss, operative time, and hospital stay, between the TLH and TAH groups. It is particularly appropriate for data that do not follow a normal distribution or when sample sizes are unequal.

Chi-square test: This test was employed for categorical data to evaluate differences in proportions, such as complication and readmission rates, between the two surgical groups. It determines whether the distribution of categories differs significantly between groups.

Fisher’s exact test: This test was used for categorical comparisons when expected frequencies in contingency tables were low. In our study, it was applied to compare the incidence of specific complications, such as bladder injury, bleeding, bowel injury, right external iliac vein injury, septic shock, and death, between the TLH and TAH groups. It is particularly beneficial for small sample sizes, ensuring accurate statistical analysis.

Statistical significance

p-Values

A threshold of p < 0.05 was used to determine statistical significance. Results with p < 0.05 indicate a significant difference between the TLH and TAH groups, particularly in outcomes such as blood loss and hospital stay. Specific comparisons, such as blood transfusion requirements, also yielded significant p-values, highlighting differences in surgical outcomes between the two groups.

CIs

CIs were used to provide a range of values that estimate the true effect size with a specified level of certainty. In this study, CIs were particularly useful in interpreting median operative times, ensuring a more robust analysis of the data. By offering numerical boundaries for comparisons between the two groups, CIs enhanced the reliability and precision of the study’s findings.

## Results

Table 1 presents a comparison of surgical histories between patients who underwent TLH (n = 56) and TAH (n = 95). The majority of patients had no prior laparoscopic surgeries, with 44 patients (78.6%) in the laparoscopic group and 75 patients (78.9%) in the abdominal group.

**Table 1:** Surgical histories and outcomes in patients undergoing TLH and TAH TAH, total abdominal hysterectomy; TLH, total laparoscopic hysterectomy

Previous laparoscopic abdominal surgeries	TLH (n = 56)		TAH (n = 95)	
	Frequency	Percentage (%)	Frequency	Percentage (%)
NA	44	78.6	75	78.9
Hernia repair	1	1.8	0	0
Complex/combined surgeries	0	0	1	1.1
Laparoscopic cholecystectomy	3	5.4	9	9.5
Laparoscopic salpingectomy	1	1.8	1	1.1
Laparoscopic appendectomy	1	1.8	4	4.2
Laparoscopic cystectomy	1	1.8	0	0
Sleeve gastrectomy	3	5.4	5	5.3
Tubal ligation	2	3.6	0	0

Among those with a history of prior surgeries, laparoscopic cholecystectomy was the most common, reported in three patients (5.4%) in the laparoscopic group and nine patients (9.5%) in the abdominal group. Other previous surgeries included sleeve gastrectomy, which was performed in three patients (5.4%) in the laparoscopic group and five patients (5.3%) in the abdominal group, as well as laparoscopic appendectomy, recorded in one patient (1.8%) in the laparoscopic group and four patients (4.2%) in the abdominal group.

Excluding CSs, Table 2 shows that 33 patients (58.9%) in the laparoscopic group and 62 patients (65.3%) in the abdominal group had no history of prior abdominal surgeries. The most common previous surgery was CS, reported in 15 patients (26.8%) in the laparoscopic group and 21 patients (22.1%) in the abdominal group. Additionally, a small number of patients had undergone laparotomy, including six patients (10.7%) in the laparoscopic group and five patients (5.3%) in the abdominal group.

**Table 2:** Surgical histories and outcomes of previous abdominal surgeries in patients undergoing TLH and TAH CS, cesarean section; TAH, total abdominal hysterectomy; TLH, total laparoscopic hysterectomy

Previous abdominal surgeries	TLH (n = 56)		TAH (n = 95)	
	Frequency	Percentage (%)	Frequency	Percentage (%)
NA	33	58.9	62	65.3
Abdominoplasty	1	1.8	3	3.2
CS	15	26.8	21	22.1
Hernia repair	1	1.8	4	4.2
Laparotomy	6	10.7	5	5.3
Total	56	100	95	100

Table 3 presents the diagnoses leading to hysterectomy. The most common indication was abnormal uterine and vaginal bleeding, reported in 30 patients (53.6%) in the laparoscopic group and 31 patients (32.6%) in the abdominal group. Uterine and endometrial conditions were also prevalent, affecting 17 patients (30.4%) in the laparoscopic group and 30 patients (31.6%) in the abdominal group. Other diagnoses included ovarian and adnexal masses, observed in three patients (5.4%) in the laparoscopic group and 10 patients (10.5%) in the abdominal group, as well as endometrial hyperplasia, which was diagnosed in two patients (3.6%) in the laparoscopic group and 13 patients (13.7%) in the abdominal group.

**Table 3:** Diagnoses leading to hysterectomy in TLH and TAH TAH, total abdominal hysterectomy; TLH, total laparoscopic hysterectomy

Diagnosis	TLH (n = 56)		TAH (n = 95)	
	Frequency	Percentage (%)	Frequency	Percentage (%)
Abnormal uterine and vaginal bleeding	30	53.6	31	32.6
Cervical conditions	2	3.6	6	6.3
Endometrial hyperplasia	2	3.6	13	13.7
Neoplasms and cancers	0	0	2	2.1
Ovarian and adnexal masses	3	5.4	10	10.5
Pelvic masses and related conditions	1	1.8	3	3.2
Pelvic organ prolapse	1	1.8	0	0
Uterine and endometrial conditions	17	30.4	30	31.6
Total	56	100	95	100

Table 4 shows that three patients (5.4%) in the laparoscopic group and six patients (6.3%) in the abdominal group were readmitted within one month after surgery. The primary reason for readmission was complications, including wound infection, which occurred in 3.2% of patients in the abdominal group. However, the majority of patients did not require readmission, with 53 patients (94.6%) in the laparoscopic group and 89 patients (93.7%) in the abdominal group remaining complication-free.

**Table 4:** Comparison of readmission reasons within one month between TLH and TAH TAH, total abdominal hysterectomy; TLH, total laparoscopic hysterectomy

Readmission within one month	TLH (n = 56)		TAH (n = 95)	
	Frequency	Percentage (%)	Frequency	Percentage (%)
NA	53	94.6	89	93.7
Abdominal collection	1	1.8	0	0
Bladder injury	0	0	1	1.1
Diaphragmatic irritation	1	1.8	0	0
Foreign body	0	0	1	1.1
Paralytic ileus	0	0	1	1.1
Injury of ureter	1	1.8	0	0
Wound infection	0	0	3	3.2

Table 5 compares intraoperative outcomes between TLH and TAH. Blood loss was significantly lower in the TLH group, with a median of 200 ml (IQR: 100-300) compared to 300 ml (IQR: 200-500) in the TAH group (p = 0.00). Similarly, hospital stays were shorter for TLH patients, with a median of two days (IQR: 1-3) versus two days (IQR: 2-3) for TAH patients, a statistically significant difference (p = 0.00). Although no blood transfusions were required in either group, the p-value remained significant (p = 0.009).

**Table 5:** Comparison of intraoperative outcomes between TLH and TAH TAH, total abdominal hysterectomy; TLH, total laparoscopic hysterectomy

Outcome	TLH	TAH	Test statistic	p-Value
Blood loss (ml)/median (IQR)	200 (100-300)	300 (200-500)	U = 5320	0
Hospital stay (days)	2 (1-3)	2 (2-3)	U = 5320	0
Blood transfusion	0	0	Fisher’s exact test	0.009

Regarding complications, Table 6 shows that most patients in both groups experienced no complications, with 52 patients in the TLH group and 88 patients in the TAH group. The overall complication rates were comparable, with four patients in the TLH group and seven in the TAH group (p = 0.959). Bladder injury occurred in two patients in each group, while septic shock and death were reported in one patient in the TLH group. Additional complications in the TAH group included three cases of bowel injury and one case of external iliac vein injury.

**Table 6:** Comparison of complications between TLH and TAH TAH, total abdominal hysterectomy; TLH, total laparoscopic hysterectomy

Complication type	TLH	TAH	Test statistic	p-Value
NA	52	88	0	0.482
Bladder injury	2	2		
Bleeding	1	1		
Bowel injury	0	3		
Right external iliac vein	0	1		
Septic shock and death	1	0		

Table 7 shows that the operative time (OR time) was slightly longer for TLH, with a median of 98.5 minutes (83.25-116.5) compared to 95 minutes (76-120) for TAH. However, this difference was not statistically significant (p = 0.633).

**Table 7:** Comparison of operative time (OR time) between TLH and TAH TAH, total abdominal hysterectomy; TLH, total laparoscopic hysterectomy

Outcome	TLH	TAH	Test statistic	p-Value
OR time, min/median (CI)	98.5 (83.25-116.5)	95 (76-120)	5,320	0.633

Regarding the number of previous laparoscopic abdominal surgeries, Table 8 shows that the majority of patients had no prior surgeries, with 43 patients in the TLH group and 75 patients in the TAH group. A smaller proportion of patients in both groups had undergone one or more previous laparoscopic procedures.

**Table 8:** Comparison of the number of previous laparoscopic abdominal surgeries between TLH and TAH TAH, total abdominal hysterectomy; TLH, total laparoscopic hysterectomy

Number of previous laparoscopic abdominal surgeries	TLH	TAH	Test statistic	p-Value
0	43	75	1.86	0.601
1	10	15		
2	1	4		
3	2	1		

Regarding the number of abdominal surgeries, including CSs, Table 9 shows that 32 patients in the TLH group and 61 patients in the TAH group had no prior surgeries. In the TLH group, 11 patients had one previous abdominal surgery, nine had two, and three had three. In comparison, the TAH group had 14 patients with one prior abdominal surgery, six with two, and eight with three. A small number of patients in each group had four or more previous abdominal surgeries.

**Table 9:** Comparison of the number of abdominal surgeries (including CS) between TLH and TAH CS, cesarean section; TAH, total abdominal hysterectomy; TLH, total laparoscopic hysterectomy

Number of previous surgeries	TLH	TAH	Test statistic	p-Value
Abdominal surgeries (including CS)			7.72	0.26
0	32	61		
1	11	14		
2	9	6		
3	3	8		
4	0	2		
5	0	3		
6	1	1		
Previous CSs				0.687
0	36	68		
1	9	10		
2	7	7		
3	3	5		
4	1	2		
5	0	2		
6	0	1		

## Discussion

The study highlights the advantages of TLH over TAH, particularly in reducing blood loss and hospital stays, even among patients with prior abdominal surgeries. TLH demonstrated comparable safety and efficacy to TAH, with no significant differences in operative time or complication rates. However, the study acknowledges certain limitations, including its retrospective design and the need for further research on long-term outcomes and cost-effectiveness.

While this study primarily focused on surgical outcomes, the role of tumor markers such as CA-125 and CA 19-9 in gynecological tumors remains significant. These markers can assist in preoperative diagnosis and risk stratification, particularly in cases involving ovarian and adnexal masses. For instance, elevated CA-125 levels may suggest malignancy, although benign conditions can also cause increases. A comprehensive preoperative assessment incorporating imaging and tumor markers is essential for accurate diagnosis and surgical planning.

Surgical outcome comparison

After surgery, significant blood loss was observed in both TLH and TAH patients. This finding aligns with existing literature emphasizing the minimally invasive nature of TLH [1], which typically results in less intraoperative trauma and reduced bleeding [2]. In this study, the median blood loss was 200 mL in the TLH group, compared to 300 mL in the TAH group. These results reinforce the advantages of laparoscopic techniques, particularly in minimizing perioperative risks for patients with prior abdominal surgeries. This consideration is especially critical for patients with adhesions, where excessive bleeding could complicate surgical outcomes.

A shorter hospital stay was noted in TLH patients, with a median of two days compared to two to three days for TAH patients. This shorter duration aligns with the benefits of minimally invasive approaches. Early ambulation and reduced postoperative pain associated with TLH likely contribute to faster recovery, benefiting patients while also reducing healthcare costs [3]. These findings further support TLH as a favorable option for minimizing hospital stays in clinical practice.

However, this study found no significant differences in operative times between TLH and TAH, with median durations of 98.5 and 95 minutes, respectively. This suggests that TLH has mitigated potential time disparities despite its technically demanding nature, which requires advanced surgical skills and experience. Importantly, prior abdominal surgeries, often associated with adhesions and altered anatomical landscapes, did not disproportionately prolong TLH procedures in this study. These results align with findings by Koroglu et al. [12], who emphasize the necessity of surgical expertise in managing complex cases. According to their research, patients with a history of previous surgeries should be handled with caution and skill to ensure optimal surgical outcomes.

Complications and safety

The study found that complication rates were low and comparable between the two groups, highlighting the safety of both approaches when performed by experienced surgeons. The overall complication rates of 7.1% for TLH and 7.4% for TAH suggest that prior abdominal surgeries do not significantly increase the risk of adverse outcomes, provided that proper preoperative planning and intraoperative vigilance are maintained. However, it is noteworthy that bowel injuries occurred exclusively in the TAH group, while septic shock and mortality were reported in a single TLH case. These findings emphasize the need for individualized surgical planning based on patient risk factors.

Bladder injuries were equally distributed between the two groups, reinforcing the importance of precise intraoperative visualization and meticulous dissection techniques. Adhesions resulting from prior CSs or other abdominal surgeries can obscure anatomical landmarks, necessitating heightened caution during surgical entry and dissection. In cases requiring conversion from TLH to TAH, a well-considered decision-making process is essential to optimize patient safety and surgical outcomes. Ultimately, ensuring patient safety and promoting optimal recovery should remain the top priority in any surgical setting.

Impact of previous abdominal surgeries

This study primarily examines the impact of prior abdominal surgeries on hysterectomy outcomes. While adhesions can increase surgical complexity, the data indicate that TLH remains a viable option for most patients. The ability to safely perform TLH in cases of previous CSs, appendectomies, and cholecystectomies highlights the growing expertise of laparoscopic surgeons. In addition to surgical proficiency, continuous technological advancements are essential in improving outcomes.

Furthermore, the findings suggest that laparotomies may present unique challenges. Patients with a history of open surgeries exhibited slightly higher complication rates, although these differences were not statistically significant. This observation aligns with previous research indicating that open surgeries often result in denser and more extensive adhesions, which can complicate subsequent laparoscopic procedures. These insights emphasize the importance of careful surgical planning and individualized approaches to optimize patient safety and surgical success.

Clinical implications

The findings of this study have significant clinical implications, particularly in promoting public health. For patients with prior abdominal surgeries, TLH presents a preferable option due to its clear advantages in reducing blood loss and minimizing hospital stays. These benefits are especially relevant within enhanced recovery after surgery (ERAS) protocols, which prioritize minimizing perioperative morbidity and facilitating early discharge. However, the decision to proceed with TLH should be individualized, taking into account factors such as the extent of adhesions, surgeon expertise, and the patient’s overall health.

The comparable operative times and complication rates observed in this study further support the feasibility of TLH in patients with previous surgeries, provided that thorough preoperative planning and intraoperative decision-making are implemented. In addition to surgical expertise, meticulous preparation plays a critical role in ensuring successful outcomes. Essential preoperative measures include imaging assessments and close collaboration with multidisciplinary teams to optimize surgical strategies and patient management.

Areas for research advancement

While this study offers valuable insights, several areas still require further investigation.

Long-Term Outcomes

Future studies should examine the long-term outcomes of TLH and TAH, including quality of life, recurrence rates of underlying conditions, and their impact on subsequent surgeries. This data would offer a more comprehensive understanding of the limitations and benefits associated with each approach.

Subgroup Analyses

Larger multicenter studies are needed to assess outcomes in specific subgroups, such as patients with multiple prior surgeries, severe adhesions, or particular comorbidities. These subgroup analyses could provide valuable insights for optimizing surgical strategies for high-risk patients.

Cost-Effectiveness

This includes factors such as operative costs, hospital stays, and postoperative recovery, which are crucial for healthcare policymakers and administrators in evaluating both the feasibility and affordability of adopting either surgical approach.

Role of Advanced Imaging

In preoperative planning, advanced imaging techniques such as MRI and 3D ultrasound are highly valuable. Further exploration is needed to assess their potential for enhancing surgical outcomes.

Training and Simulation

Early and high-quality training in laparoscopic surgery is crucial for improving outcomes. Additionally, simulations can enhance surgeons’ understanding of the procedure, providing valuable insights that contribute to successful task completion.

Patient-Reported Outcomes

Future studies should include patient-reported outcomes, such as pain, satisfaction, and return to daily activities, to offer a more comprehensive assessment of the impact of TLH and TAH on patient well-being. By addressing these areas, future research can build on the findings of this study and further refine surgical strategies for patients undergoing hysterectomy. The ultimate goal is to enhance patient safety through optimized outcomes and advance the field of gynecological surgery.

## Conclusions

This study highlights the significant advantages of TLH over TAH, particularly in reducing intraoperative blood loss and hospital stays, even in patients with prior abdominal surgeries. TLH was effective in minimizing perioperative risks without significantly increasing operative time or complication rates, which remained comparable between the two groups. Despite the technical challenges posed by adhesions and altered anatomy from previous surgeries, TLH proved to be a safe and feasible option when performed by experienced surgeons. These findings underscore TLH’s role as a preferred approach for improving patient outcomes and facilitating quicker recoveries, especially within ERAS protocols.

The comparable safety profiles of TLH and TAH highlight the importance of individualized surgical planning, taking into account patient-specific factors such as the extent of adhesions and overall health. While TAH may still be necessary in certain cases, the results suggest that advancements in laparoscopic techniques and surgical expertise enable TLH to be successfully performed in increasingly complex scenarios. The study also emphasizes the need for careful preoperative preparation, including imaging and interdisciplinary collaboration, to optimize surgical outcomes. Future research should focus on refining laparoscopic methods and exploring strategies to improve outcomes for patients with a history of extensive abdominal surgeries. TLH is a safe and effective option for patients with prior abdominal surgeries, offering reduced blood loss and shorter hospital stays compared to TAH. The decision between TLH and TAH should be individualized, considering factors such as the extent of adhesions, surgeon expertise, and patient health. Further research is needed to optimize surgical outcomes in complex cases.
